# Comprehensive human amniotic fluid metagenomics supports the sterile womb hypothesis

**DOI:** 10.1038/s41598-022-10869-7

**Published:** 2022-04-27

**Authors:** HanChen Wang, Gui Xiang Yang, Yuxiang Hu, Patricia Lam, Karan Sangha, Dawn Siciliano, Anne Swenerton, Ruth Miller, Peter Tilley, Peter Von Dadelszen, Shirin Kalyan, Patrick Tang, Millan S. Patel

**Affiliations:** 1 Department of Medical Genetics, University of British Columbia, Vancouver, BC Canada; 2 Department of Physiology, McGill University, Montreal, QC Canada; 3 Department of Obstetrics and Gynaecology, University of British Columbia, Vancouver, BC Canada; 4 CureImmune Therapeutics Inc., Vancouver, BC Canada; 5 Center for Gene Therapy, Abigail Wexner Research Institute, Nationwide Children’s Hospital, Columbus, OH USA; 6 Department of Pathology and Laboratory Medicine, University of British Columbia, Vancouver, BC Canada; 7 British Columbia Centre for Disease Control, Vancouver, BC Canada; 8 Contextual Genomics Inc., Vancouver, BC Canada; 9 Department of Women and Children’s Health, School of Life Course Sciences, King’s College London, London, UK; 10 Division of Endocrinology and Metabolism, Department of Medicine, University of British Columbia, Vancouver, BC Canada; 11 Department of Pathology, Sidra Medical and Research Center, Doha, Qatar; 12 BC Children’s Hospital Research Institute, University of British Columbia, Vancouver, BC Canada; 13 Department of Medical Genetics, University of British Columbia, 4500 Oak St., Rm. C234, Vancouver, BC V6H 3N1 Canada

**Keywords:** Translational research, Diseases, Molecular medicine, Classification and taxonomy

## Abstract

As metagenomic approaches for detecting infectious agents have improved, each tissue that was once thought to be sterile has been found to harbor a variety of microorganisms. Controversy still exists over the status of amniotic fluid, which is part of an immunologically privileged zone that is required to prevent maternal immune system rejection of the fetus. Due to this privilege, the exclusion of microbes has been proposed to be mandatory, leading to the sterile womb hypothesis. Since nucleic acid yields from amniotic fluid are very low, contaminating nucleic acid found in water, reagents and the laboratory environment frequently confound attempts to address this hypothesis. Here we present metagenomic criteria for microorganism detection and a metagenomic method able to be performed with small volumes of starting material, while controlling for exogenous contamination, to circumvent these and other pitfalls. We use this method to show that human mid-gestational amniotic fluid has no detectable virome or microbiome, supporting the sterile womb hypothesis.

## Introduction

Bacterial or viral invasion of the amniotic cavity has been associated with fetal loss, birth defects, fetal anemia, preterm premature rupture of membranes, preterm labor and birth, and maternal mortality^1–5^. Intact chorioamniotic membranes are essential to prevent invasion, as the majority of mothers will develop microbial invasion of the amniotic cavity (MIAC) after membrane rupture^6,7^. This observation, along with the inevitability of preterm birth after MIAC, illustrates that the amniotic cavity has a limited ability to combat active infection. This has been proposed, in part, to be due to the maternal immunologic privilege afforded the fetus, which must be maintained to prevent rejection of the pregnancy by the maternal immune system. Both the immune privilege and the intolerance for MIAC have led to the proposal that amniotic fluid must be sterile, known as the sterile womb hypothesis. Evidence documenting this sterility was first provided by Escherich^8^.

Addressing this hypothesis and understanding the full impact of infection in pregnancy requires development of methods capable of the unbiased detection of all potential pathogens in samples from affected pregnancies. To this end, a variety of methods have been developed with various degrees of sensitivity and specificity, including: Degenerate Oligonucleotide Primed PCR^9^, Virus Discovery based on cDNA Amplified Fragment Length Polymorphism (VIDISCA)^10,11^, the Virochip^12^, Comprehensive serological profiling^13^, or large scale multiplexed qPCR^14^. Each of these methods has different limitations such as low sensitivity (up to 10^6^ genome copies/mL) in biological fluids for some, requirement for large scale and ongoing assay development, or lack of flexibility. Recently, massively parallel metagenomic sequencing methods have been used to suggest the presence of microbial nucleic acid in amniotic fluid^15,16^, placenta^17^, umbilical cord blood^18^ and meconium^19,20^. However, other studies have disputed these findings in placenta^21,22^ and amniotic fluid^23–26^.

Several methodologic problems have plagued metagenomic analyses, and these are particularly acute with cell poor fluids such as amniotic fluid, hampering efforts to determine whether or not amniotic fluid is sterile and if not, to define its microbiome^27^. These problems include the inapplicability of common library construction methods to sub-nanogram amounts of starting material^28^; lack of method sensitivity to all DNA and RNA viruses, bacteria and protozoa^16^; lack of comprehensive pathogen databases free of human sequence contamination^29^; microbial nucleic acid contamination introduced from sample handling, buffers and reagents^30,31^; within species variability, especially for viruses, which hampers accurate detection^32,33^; similarities of some microorganism sequences to human resulting in decreased sensitivity after filtering of human sequences; and a general lack of rigor in distinguishing between a single read partial sequence match versus reliable identification of a microorganism’s nucleic acid. Here we report development of rigorous criteria and methodology to address these challenges and apply these to the question of whether amniotic fluid from healthy pregnancies contains pathogen or other microbial nucleic acid. We find no reliably detectable microorganism nucleic acid in our samples, a result consistent with the sterile womb hypothesis.

## Methods

### Amniotic fluid collection

Samples were obtained from healthy women in mid-gestation with a maternal serum AFP or hCG of greater than 2 and less than 3.5 multiples of the median who chose to proceed to amniocentesis and who were consented after their genetic counseling session at BC Women’s Hospital, Vancouver, Canada. Institutional Research Ethics Board approval was obtained (#H07-00353) and informed consent obtained from all participants. Amniotic fluid was collected under sterile conditions as two 1.0 mL aliquots, frozen in liquid nitrogen within 60 s of removal from the participant and stored frozen at −80 °C until analysis. Only samples with normal karyotype where the pregnancy proceeded to term birth and resulted in a healthy infant were retained. Samples from pregnancies affected by congenital cytomegalovirus (CMV) diagnosed by PCR and *Fusobacterium nucleatum*^34,35^ and *Toxoplasma*, both diagnosed by culture, were obtained from the Maternal Fetal Medicine Biobank (MFMB) in Barcelona, Spain. After obtaining informed consent, MFMB samples were collected as 2.0 mL aliquots and frozen within 1 h of collection at −80 °C until analysis.

### Library construction

DNA and RNA were isolated from 200 μl of amniotic fluid using QIAamp MinElute Virus spin kit (Qiagen, Tarrytown, MD) except where indicated. Water controls consisted of lot-matched Qiagen extraction buffer. cDNA was prepared with half the resulting eluate to capture RNA viruses using ThermoFisher SuperScript VILO Master Mix (ThermoFisher, Carlsabad, CA). Second strand synthesis was not performed as we found it resulted in a reduced number of detectable reads for DNA viruses (Suppl Table 1). DNA and cDNA for each sample were pooled and concentrated on a Zymo Clean and Concentrate column (Zymo Research, Irvine, CA). Genome sequencing libraries were prepared from the resulting concentrate according to manufacturer’s instructions using the PicoPLEX library construction kit with barcoding (Rubicon Genomics, Ann Arbor, MI) and purified using AMPure beads (Beckman Coulter, Brea, CA). The numbers of spiked targets recovered at each stage of library preparation is shown in Suppl Table 2. The standard Illumina library preparation methods and the Nextera library kit (Epicentre Biotechnologies, Madison, WI) were not found to work for our samples due to the small amount of starting material (often < 1 ng), although this limitation has recently been addressed^36^.

### Database construction

To improve pathogen detection, we constructed reference viral, bacterial, fungal, and protozoan genome databases that incorporated sequences from multiple sources. For the viral database, a combination of NCBI Virus and Viroid Genome Databases^37^, European Nucleotide Archive^38^, the Actinobacteriophage Database^39^, and Subviral RNA Database^40^ were used. There are 11,624 viral genomes in the resulting database, consisting of 7392 species. Some species, due to variation in their genomes or lack of a complete genomic assembly, have more than one representative sequence in the database. These include Rotavirus A, Hepatitis B virus, Human immunodeficiency virus 1, Dengue virus, West Nile virus, Zaire ebolavirus and Porcine circovirus 2. For the bacterial database, a combination of NCBI RefSeq^41^, NCBI Genbank^42^ and ENSEMBL^43^ assembly sequences were used with non-chromosomal, contigs and non-genomic sequences manually removed. There are 21,685 bacterial genomes or scaffolds in the database. For the fungal and protozoan database, a combination of NCBI RefSeq^41^ and NCBI Genbank^42^ assembly sequences were used with non-chromosomal and non-genomic sequences removed. There are 1043 fungal and 250 protozoan species in the database. Contaminating human or human-like sequences were removed from databases as follows: After analyzing 100 human samples from various tissue sources, certain viruses, protozoa and intracellular bacteria were consistently detected in all samples. These reads always comprised many identical reads mapping to specific regions of the organism in question. BLAST searches with these sequences revealed similarity to human; thus, we filtered our reference databases for sequences with a minimum alignment to human of 60 bp, with a maximum of 15% gaps, 15% mismatches, or 30% gaps plus mismatches. Microorganism sequences meeting these criteria were flagged and removed from our pathogen databases. Suppl Tables 3–6 contain a complete list of removed sequences.

### Bioinformatics

**Figure 1:**
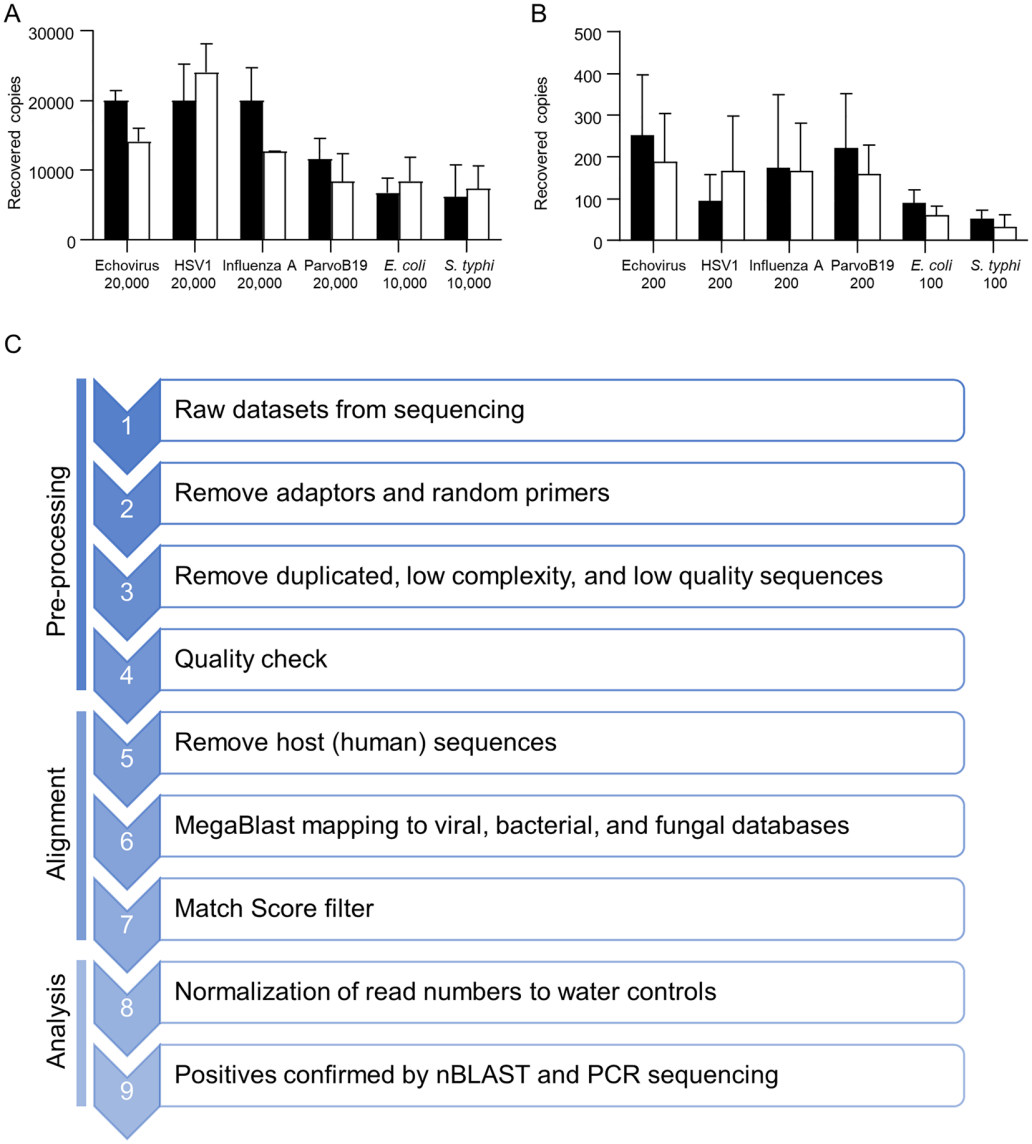
Viral and bacterial genome copy yield from human amniotic fluid by extraction method. (**A,B**) Recovered copies for various pathogens and spike levels as indicated on the x axis for the MinElute (black bars) and PureLink (white bars) columns. Error bars represent SD for 3 experiments. (**C**) Overview of the metagenomic method used.

Genome sequencing libraries without size fractionation were subjected to 100 bp or 125 bp paired end sequencing on Illumina HiSeq 2000 or 2500 machines at the McGill University and Génome Québec Innovation Centre. After QC, libraries were split and sequenced in stoichiometric proportions in different lanes on different flow cells to reduce the known stochastic lane bias for sequencing errors. The resulting sequence reads were filtered as follows: Adaptors introduced during sequencing were removed using Cutadapt 1.10^44^ with the adaptor sequences designated as AGATCGGAAGAGC and AGATCGGAAGAGCACACGTCTGAACTCCAGTCACCGATGTATCTCGTATGCCGTCTTCTGCTTG. Quality control was performed with PRINSEQ^45^ to remove duplicated, low complexity, and low quality sequences. Further filtering of low-quality sequences was done with a custom Python script that removed reads that failed the Illumina Chastity filter, had more than 20 consecutive identical bases, contained at least one “N”, had less than two-thirds of the bases with a quality of >  = 30 in the first half, or were less than 70 bp long. The script also removed the B-tail. These steps are summarized in Fig. 1C.

Human sequence reads were identified using bowtie2 v 2.1.0^46^ with the sensitive mode to a human genome database containing the UCSC’s hg19 human reference genome^47^, human mitochondrial DNA and ribosomal RNA sequences^41^. After removing the reads that were mapped to human, the remaining reads were remapped to the LSU and SSU SILVA databases^48^ to further remove ribosomal DNA sequences and to the human genome database using BMTagger^49^ to remove any remaining human sequences. After filtering, each read was mapped to our 3 microorganism genome databases using the nucleotide search tool MEGABLAST v2.2.30 + ^50^. We tested the number of processors required to perform MEGABLAST and found the optimum number to be 7 with only a small additional gain with 8 or more processors (Suppl Fig. 1). Since one processor from each node often is down, we performed MEGABLAST with 7 processors per sample, keeping the eighth as a backup. Minimum alignments were set at 60 nucleotides. An in-house python script was used to eliminate reads that had more than 15% mismatches, 15% gaps, or 30% mismatches and gaps after alignment; gaps and mismatches were permitted to account for the diversity of viral reads. After MEGABLAST, the organisms with the greatest length of homology were first retained and among these, the GI or Accession number with the greatest identity (highest E score) was identified as the top match. Due to systematic differences in sequence complexity between databases, the viral database was interrogated first, with all unmatched reads then mapped to the bacterial database, followed by matching remaining unmatched reads to the fungal/protozoan database. This process resulted in some matches that eventually were found to be of human origin; thus, strategies to reduce the number of false positives were pursued.

To further eliminate human reads with some homology to microorganisms, a match score was calculated for each read using the formula:

$$ MatchScore\, = \,\left( {\% \, bases \, aligned \, to \, organism\, \times \,Alignment \, Length} \right) \, - (\%\,bases \, aligned \, to \, human\, \times \,Alignment \, length) $$

Reads with Match scores of 0 or higher were retained and the MEGABLAST results were exported in Excel format. Then, using NCBI Taxonomy’s GI number to Taxon ID and Accession number to Taxon ID references, the number of matching reads for each organism detected were reported at the species, genus, and family levels^51^.

### Water normalization score (WNS)

We processed water through our library pipeline and sequenced 5 water control libraries, only analyzing reads where both members of the read pair matched to the same species. For all samples, the total read count per species (or genus) was expressed as a fraction of the total number of sequenced reads passing quality filters from that sample and this was divided by the fraction seen in water controls to give a Water Normalization Score:

$$ Water\;Normalization\;Score\left( {WNS} \right) = {{\frac{{\# \;of\;reads\;matching\;an\;organism\;in\;a\;sample}}{{total\;\# \;of\;sequence\;reads\;for\;that\;sample}}} \mathord{\left/ {\vphantom {{\frac{{\# \;of\;reads\;matching\;an\;organism\;in\;a\;sample}}{{total\;\# \;of\;sequence\;reads\;for\;that\;sample}}} {\frac{{\# \;of\;reads\;for\;the\;same\;organism\;in\;all\;water\;controls}}{{total\;\# \;sequence\;reads\;in\;all\;water\;controls}}}}} \right. \kern-\nulldelimiterspace} {\frac{{\# \;of\;reads\;for\;the\;same\;organism\;in\;all\;water\;controls}}{{total\;\# \;sequence\;reads\;in\;all\;water\;controls}}}} $$

Thus, a higher WNS indicates a greater fraction of reads in a sample than in water controls.

### Criteria for diagnostic reads—development of bioinformatic Koch’s postulates

Not infrequently, we found small amounts of human sequence contamination in pathogen reference genomes, indicating that a single well-matched pathogen read may not constitute sufficient evidence for the presence of an organism’s nucleic acid. To establish a more rigorous evidential basis, we defined a ‘diagnostic read’ as the minimum set of data required to infer the presence of an organism’s nucleic acid in a sample, with the criteria as follows:

- Both paired end reads must match to the same species
- At least 3 separate, discrete paired end reads must match to the same species
- Match score > 0 for all reads
- Water Normalization Score > 1.0 for organism

A read was marked as discrete when, after computationally linking the forward and reverse reads, there was less than 50 bp of sequence overlap with any other connected read pairs matching the same species in that sample.

### Confirmation of diagnostic reads

After bioinformatic detection of a pathogen nucleic acid, we developed a droplet digital PCR assay specifically for that pathogen and used this to titre the organism using a separate aliquot of amniotic fluid of the sample in question. BioRad (Hercules, California) reagents, droplet maker and QX200 reader were used for these assays. In addition or as an alternative to confirm the bioinformatic result, we used pathogen-specific primers to perform PCR sequencing of the resulting fragment and used BLASTn to confirm pathogen identity. For bacteria, a species-specific marker gene was used but where unavailable, we performed bacterial 16S rDNA PCR using the 27Fmod and 534R or 907R primers, subcloned the fragments and sequenced each clone individually to resolve or confirm bacterial identity at the genus level. Primer sequences and annealing temperatures used are shown in Suppl Table 7.

### Principal component analysis

Principal component analysis was performed for the water and amniotic fluid samples using Log2-transformed Water Normalization Scores (WNS).

Computational resources for this study were generously provided by WestGrid, the western Canada supercomputing grid, using the Jasper, Breezy and Cedar servers running Linux 1.08. Infection control standards and procedures for Containment Level II were followed at all times under HPTA Registration No. R-10-000455.

The authors confirm that all methods were carried out in accordance with relevant guidelines and regulations.

## Results

We first sought to determine which nucleic acid isolation method was most efficient for recovering DNA and RNA viruses from amniotic fluid. We also wanted to assess unenveloped and enveloped, segmented and unsegmented, long and short, and single- and double-stranded viruses, which has not been addressed in recent literature to the best of our knowledge. Since input volumes for library preparation from small amounts of nucleic acid is very limited, we constrained our evaluation to methods with low elution volumes. To this end, we initially compared 3 silica-based viral RNA/DNA isolation methods with standard Trizol or Phenol extraction for the smallest known human pathogen, hepatitis delta virus (HDV). We also assessed one of the largest viruses, human herpesvirus 5, commonly known as Cytomegalovirus (CMV) (Suppl Fig. 2A, B). One silica column, Qiagen MinElute, clearly outperformed the rest. Later, another silica-based column (ThermoFisher Purelink) became available and its performance was compared for various levels of input virus or bacteria (Fig. 1A,B). Overall, the Qiagen MinElute viral RNA/DNA column performed slightly better for both viruses and bacteria and was used in the remainder of our studies.

To comprehensively define a microbiome in amniotic fluid, we developed a metagenomic protocol as detailed in the Methods; an overview is provided in Fig. 1C. As an initial assessment of whether our method could detect a wide size range of microorganisms, we spiked 3 exogenous agents (*E. coli*, enterovirus B and HDV) into control amniotic fluid and determined whether sequencing reads could be recovered to identify these agents. All 3, even the smallest known human pathogen, HDV, were clearly identified from amniotic fluid at low spike levels (< 10^3^ genome copies/mL) (Suppl Fig. 2C).

**Figure 2:**
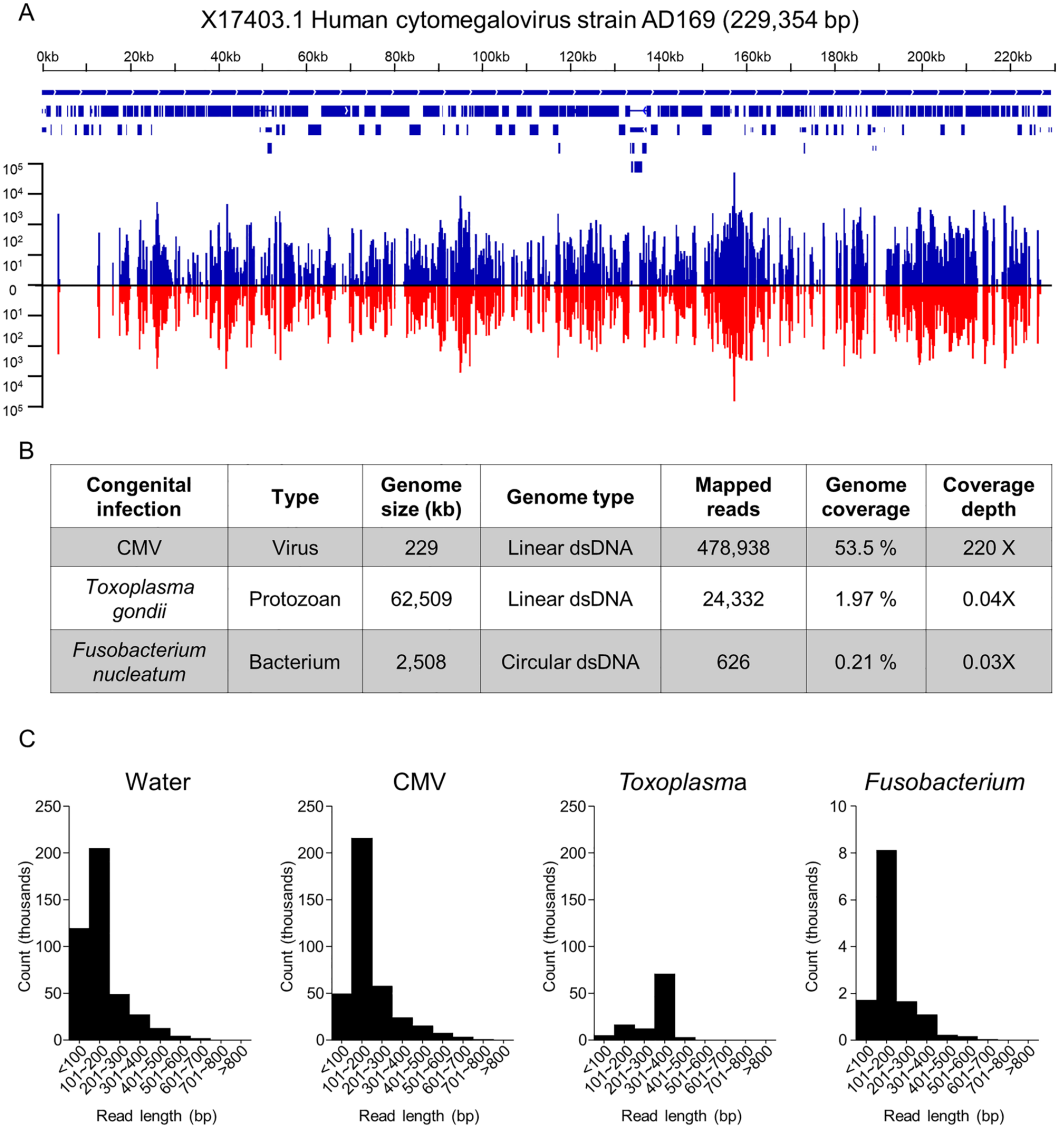
Detection of known infections. (**A**) Genomic graph illustrating the coverage and depth of CMV in the CMV-positive amniotic fluid sample (against reference CMV genome NC_006273.2). Top: gene location and genomic feature annotations from RefSeq. Bottom: number of reads mapped to genome. Blue lines above and red lines below the horizontal line indicate forward and reverse reads, respectively. The graph is generated using Bedfile and genomic snapshots from IGV. (**B**) Table showing genome coverage statistics for CMV, *F. nucleatum*, and *T. gondii*. (**C**) Histograms showing insert lengths (specifically, mapped paired end read separation distance) in water (left), CMV infection (middle left) and *F. nucleatum* infection (middle right) and *T. gondii* infection (right).

We next sought to determine if our method could detect known infections of the amniotic cavity. Libraries were prepared from amniotic fluid from three patients confirmed by culture and/or PCR to be infected with CMV, *Fusobacterium nucleatum* or *Toxoplasma gondii*. These viral, bacterial, and protozoan congenital infections were well detected with 478,938 reads for CMV with 53.5% genome coverage, 626 reads for *Fusobacterium* with 0.21% genome coverage, and 24,332 reads for *Toxoplasma* with 2% coverage of its much larger genome (Fig. 2A,B). No superinfecting organisms or co-infections were detected in our analyses. We plotted histograms showing the distances between paired end reads (that is, the calculated fragment insert lengths) (Fig. 2C) and found no systematic differences between water controls and infected amniotic fluid samples, which suggested no read length bias according to target. This is important because if some targets were incorporated at much longer read lengths it could introduce an amplification bias during library preparation. This would create a representational bias in the finished library that would be expected to vary according to the mix of organisms in the starting sample. The read length histograms do not show evidence of this effect.

**Figure 3:**
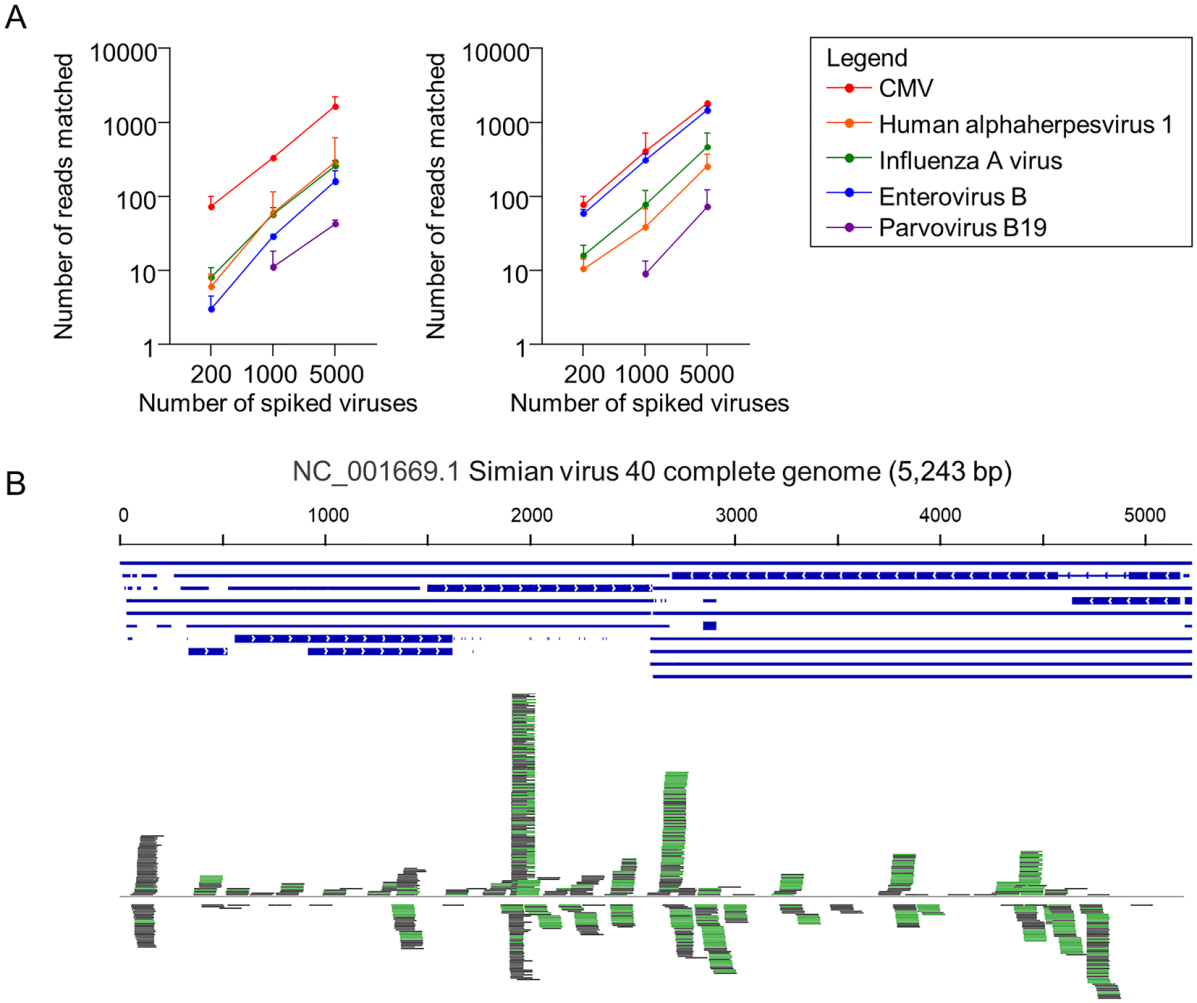
Detection of spiked viruses in human amniotic fluid. **(A)** Dot plot comparing number of reads identified from five spiked viruses at 3 different levels using our method (left panel) and IDSeq (right panel). (**B**) The 5.2 kb SV40 genome backbone is illustrated horizontally, with forward reads only shown above and below in green and grey.

To assess our method’s suitability for defining an amniotic fluid virome, we determined whether viruses with different characteristics could be detected. We spiked viral culture supernatant containing 5000 genome copies (gc), as assessed by real time quantitative PCR, from five different viral species into two different control amniotic fluids (Table 1). After sequencing, filtering, and searching against our custom databases, all viruses were found to be detectable with a range of 21 to 787 diagnostic reads (see “Methods” for a definition of this term). To search for a lower bound where test viruses could be detected, we repeated the spiking experiments with 1000 and 200 gc, respectively, for each of the five viral species. Each experiment was performed twice using a different control amniotic fluid. The relationship between read numbers matching the spiked viruses and total number of virions spiked (targets) is shown in Fig. 3A (left panel).

**Table 1:** Diagnostic viral read numbers after spiking 5000 genome copies of 5 different cultured viruses into 200 μl human amniotic fluid.

Number of genome copies	Species	Nucleic acid	Genome size (kb)	Alignment length (mean bp)	Diagnostic read number (mean ± SD)
5000	Human alpha-herpesvirus 1 (HSV1) Taxon ID: 10298	Linear dsDNA	152	1158	130 ± 150
5000	CMV Taxon ID: 10359	Linear dsDNA	236	8787	787 ± 272
5000	Influenza A virus Taxon ID: 11320	Linear ssRNA	14	507	118 ± 21
5000	Enterovirus B Taxon ID: 138949	Linear ssRNA	7.4	813	248 ± 82
5000	Parvovirus B19 Taxon ID: 10798	Linear ssDNA	5.6	654	21 ± 3

*ds* double stranded, *ss* single stranded.

To assess how our taxonomic assignment pipeline compared with a more established pipeline, we also analyzed our spiked samples using IDSeq^52^, kindly provided as a free online service at: https://idseq.net/. We found both methods provided comparable levels of detection and correct taxonomic assignment, with the exception of Enterovirus, which IDSeq identified more robustly (Fig. 3A, right panel). To assess the evenness of genome coverage with our library preparation method, the 5.2 kb circular double stranded DNA virus, SV40, was spiked in at much higher levels. The resulting genome coverage is shown in Fig. 3B, demonstrating uneven coverage of a small genome even at a high viral titre. These results demonstrate that our method has a lower limit of detection of 1000 gc/mL in amniotic fluid for all but one virus, the 5.2 kb Parvovirus B19, for which the detection limit was 5000 gc/mL. Both of these levels are at or below the level at which viruses generally cause disease in pregnancy^53–55^.

To account for the frequent presence of microbial and viral nucleic acid sequences in water/buffer controls, we developed the Water Normalization Score (WNS). A WNS of 0.25 or greater (range 0.25–8.46) was seen for each spiked virus in our low-level spike using 200 virus particles, indicating this score should be used for experiments requiring maximal sensitivity. As expected, in higher level spiking experiments with 5000 virus particles, we obtained higher WNS scores (range 7.8–362.8). To determine the variability in WNS, we calculated WNS for individual water sample datasets, comparing each one to rest of the water controls, and found the mean (± SD) was 0.85 (± 1.63) with a range of 0–7.0. Since competitor nucleic acid as low as 20 pg has been shown to suppress the majority of contaminant reads^56^, we used a WNS of 1.0 as a cut-off, with scores above 1.0 indicating a positive finding. Using this cut-off, 33%, 100% and 100% of our 200, 1000 and 5000 virus particle spikes, respectively, would be classified as positive, suggesting this is a fairly conservative threshold.

Having validated a method that is reasonably sensitive, specific, and robust to a variety of pathogens, we sought to test the sterile womb hypothesis by sequencing amniotic fluid from healthy pregnancies, which were sampled due to abnormal serum screening results. To this end, we subjected 17 samples of amniotic fluid, collected in mid-gestation from healthy women who went on to deliver healthy, karyotypically normal infants at term, to our sequencing and analysis pipeline. The characteristics of the study population are shown in Table 2. Raw read numbers for all 17 samples were 41.8 million ± 9.9 million reads (mean ± SD). Read numbers at different stages of filtering are shown in Suppl Table 9.

**Table 2:** Characteristics of the study population (n = 17).

Characteristic	Mean ± SD
Maternal age at delivery (years)	34.6 ± 4.7
Height (m)	1.68 ± 0.07
Weight (kg)	65.6 ± 10
Amniocentesis (weeks of gestation)	18.5 ± 1.8
Delivery (weeks of gestation)	39.3 ± 1.8

All 17 infants were karyotypically normal on amniocentesis and healthy at birth with normal birth weights.

We attempted to falsify the sterile womb hypothesis by identifying microbial nucleic acid using three methods: (1) through microbial identification using our pipeline to process metagenomic data, (2) through microbial identification using the IDSeq pipeline to process the same metagenomic data, and (3) by the traditional method of direct 16S rDNA PCR amplification on amniotic fluid, subcloning and Sanger sequencing of subclones. Confirmation of positive bioinformatic findings was then attempted by PCR sequencing.

**Figure 4:**
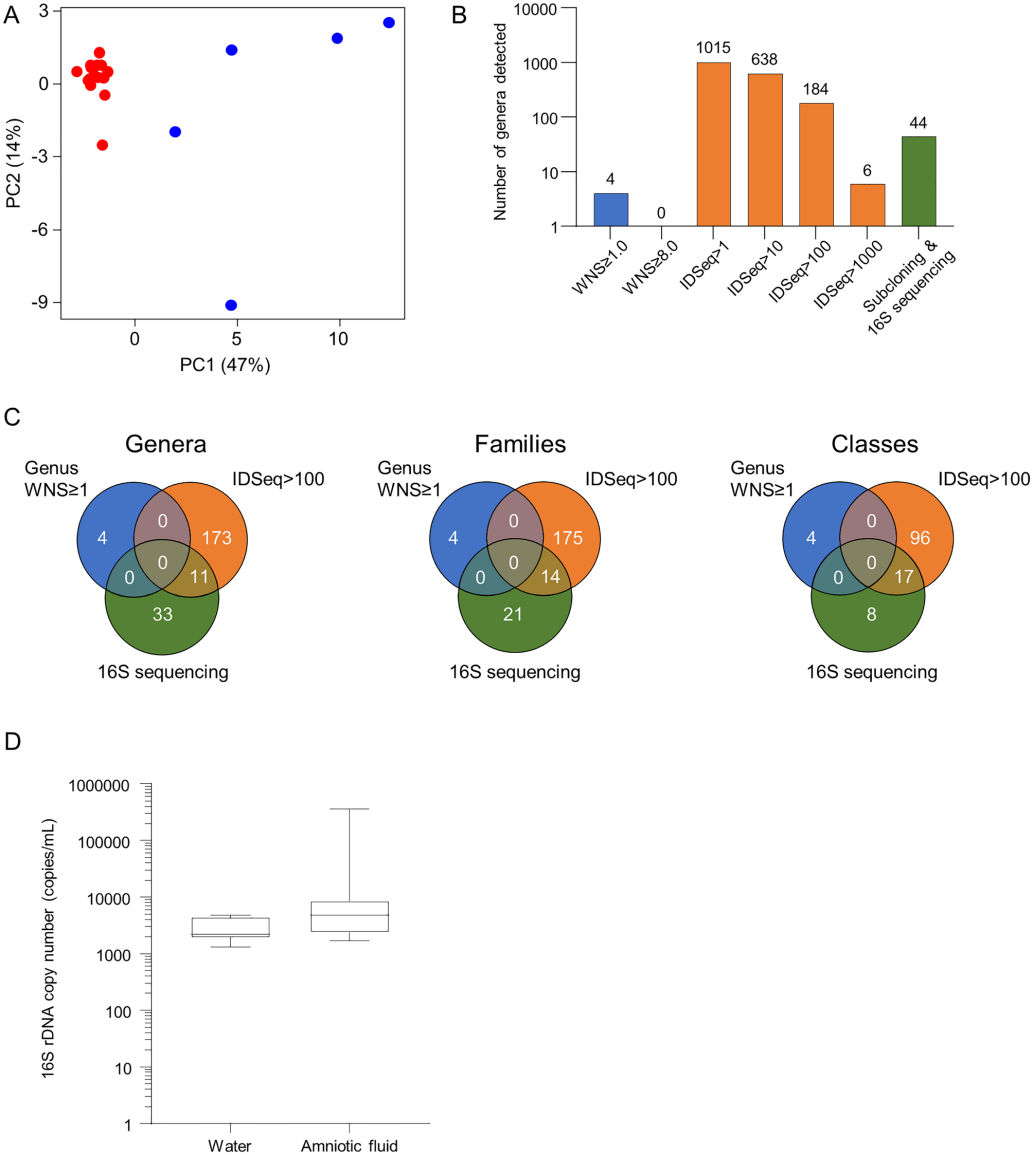
Microbial detection varies by method used. (**A**) Principal component analysis illustrating sample variance between water (blue) and amniotic fluid (red). (**B**) Bar graph showing the number of genera detected using our method (blue), IDSeq at different thresholds (orange), and 16S sequencing (green). (**C**) Venn diagrams comparing the number of genera, families, and classes detected in amniotic fluid samples using our method (blue), IDSeq (orange), and 16S sequencing (green). (**D**) EvaGreen 16S rDNA droplet digital PCR box plot comparing total volume of bacterial rDNA signatures in water controls and amniotic fluid (p > 0.05 for difference).

Principal components analysis (PCA) of filtered sequence data from amniotic fluid samples and water samples showed tight clustering of the amniotic fluid samples suggesting they had reduced variability, which would be unexpected if a diverse microbiome were present (Fig. 4A). We looked for organisms in our amniotic fluid samples with the expectation that true positives should be identified by all 3 detection methods used. We first looked at the number of genera identified by the 3 methods and found this varied widely (Fig. 4B). As expected, our method had the lowest levels of detection due to more stringent diagnostic read calling criteria.

We also looked at whether common genera were identified across the 3 methods and found no consistent data (Fig. 4C). Although there were 11 common genera identified by IDSeq and 16S sequencing, BLASTn matching of 16S sequences from genera with 100 or more reads identified by IDSeq with the 16S rDNA sequences identified did not result in any matches with complete identity so we concluded the 2 methods were identifying different species (Suppl Fig. 2D). When filtering for genera with 97% or greater homology, the closest species identified by IDSeq were seen in 3 samples: *Methylorubrum extorquens, Methylobacterium radiotolerans, Acidovorax temperans and Pelomonas saccharophila* (Suppl Table 8). Because each of these is commonly found in soil and/or water and well represented in water controls, we presumed they were environmental contaminants and did not pursue them further. For reference, the WNS ranges from our method for these 4 species in amniotic fluid were 0.00–0.26, not detected, 0.000–0.005, and not detected, respectively whereas the WNS ranges for these 4 species in water controls were 0.01–1.38, 0.0–2.4, 0.02–0.64, and not detected, respectively.

**Figure 5:**
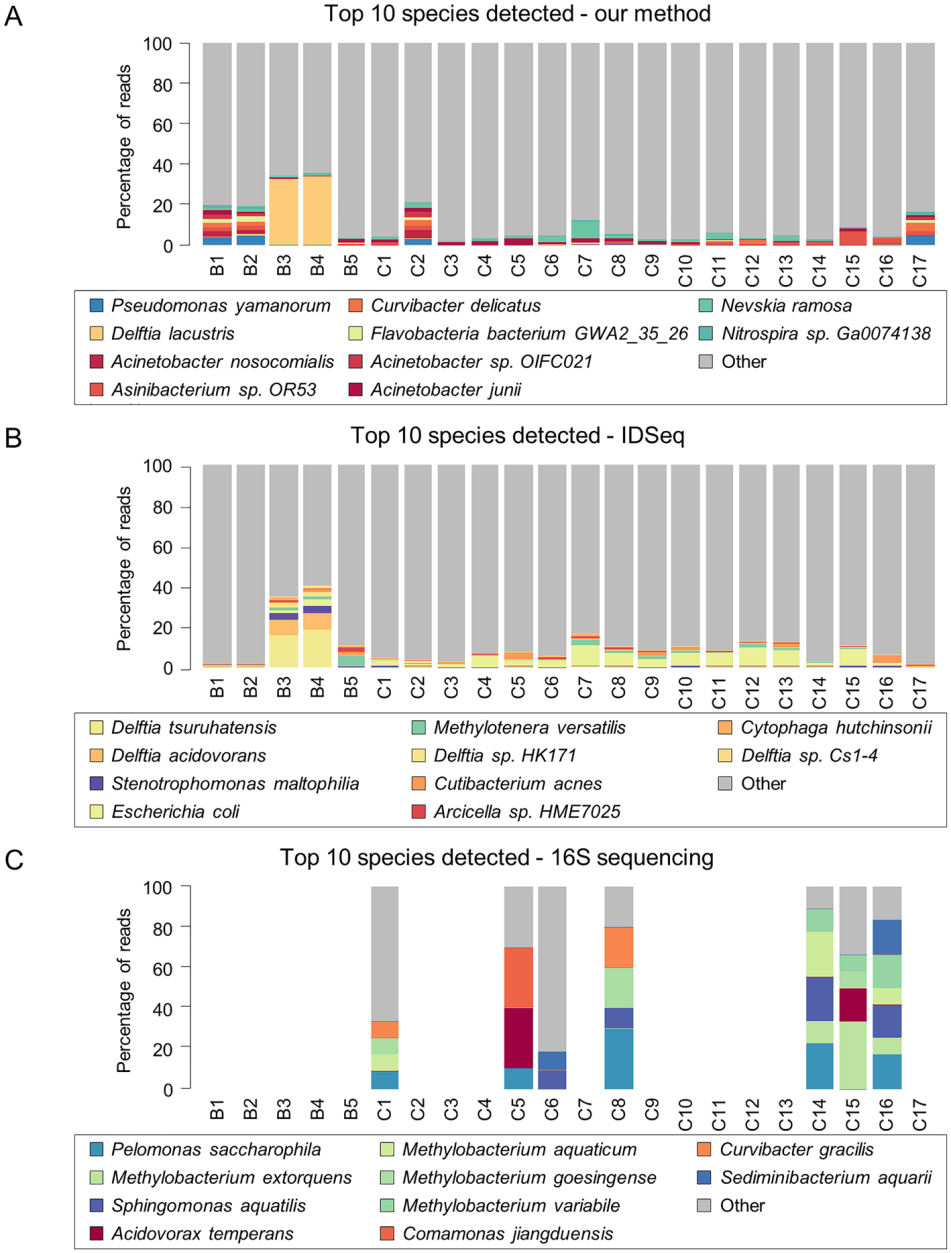
Profiles of all species detected using our method, IDSeq, and 16S sequencing. (**A–C**) Blot plots show the percentages of reads mapped to top 10 most abundant species using (**A**) our method, (**B**) IDSeq, and (**C**) 16S PCR subcloning and sequencing. Species were inferred from 16S sequence data by 100% sequence matches to the organisms indicated.

We next tried to look at the most abundant species identified by all 3 detection methods. We found these varied entirely by the detection method used (Fig. 5). This finding suggested stochastic effects were at play such that noise was the primary determinant of a positive result. The observation that there was no consistently identifiable microbiome was reinforced by the lack of an amplifiable 16S rDNA band in 41% (7/17) of our amniotic fluid samples (Fig. 5C), despite using different 16S primer sets. As a control for sequencing run-specific effects on microbial detection, 2 water controls and 2 amniotic fluid samples that were sequenced in multiple lanes on different flow cells were compared. Minimal run-to-run variation in the spectrum of nucleic acid identification was observed in both sample types (Suppl Fig. 3).

We next focused on those species detected by our pipeline that fulfilled all of our criteria, including a WNS of 1.0 or higher, and identified reads corresponding to five taxons in two samples (Table 3). To follow up on these, we computationally extracted all matching reads and used BLASTn to confirm the matches, resulting in elimination of one organism due to mis-assignment and another due to no clear match being detected. Confirmation of the three remaining organisms was attempted and none of the species could be confirmed by organism-specific PCR.

**Table 3:** Positive bioinformatic hits from amniotic fluid.

Taxon (ID)	Positive samples	No. diagnostic reads	WNS	nBLAST result	PCR result
*Autographa californica multiple nucleopolyhedrovirus *(Taxon ID: 307456)	C1, C4	3, 4	2.0, 2.5	Confirmed	Negative
*Candida tropicalis *(Taxon ID: 5482)	C1	3	8.1	Confirmed	Negative
*Cyberlindnera jadinii *(Taxon ID: 4903)	C1	36	3.6	Confirmed	Negative
*Stramenopiles sp. TOSAG41-1 *(Taxon ID: 1735744)	C4	10	1.5	PhiX174 match	ND
*Trichoderma koningii *(Taxon ID: 97093)	C1	4	10.8	Distant match, no clear result	ND

*WNS* water normalization score, *ND* not done.

An amniotic fluid virome was not identified in our analyses. We identified *Autographa californica multiple nucleopolyhedrovirus*, an insect-specific *Baculovirus*, in two samples but this was not confirmed on multiple PCR attempts with two different primer sets. The matching reads from the Autographa genome were eventually mapped to a fragment of the beta-galactosidase gene commonly found in cloning vector sequences, indicating this identification was a false positive.

To screen for bacteria that may have been missed because their genomes were not in the IDSeq or our custom reference databases, we screened for bacterial abundance using quantitative 16S rDNA droplet digital PCR with EvaGreen dye (Fig. 4D). Overall, the results demonstrate no significant difference (Mann Whitney U test, Z = 1.34; p > 0.05) in 16S rDNA copy number between amniotic fluid from healthy pregnancies and water controls.

We conclude that neither a virome nor a microbiome could reliably be demonstrated in healthy mid-gestation human amniotic fluid.

## Discussion

Using stringent collection techniques mandating less than 60 s between patient extraction and freezing in liquid nitrogen, we found amniotic fluid sampled from mid-gestation in 17 healthy pregnancies to be free of viral and protozoan nucleic acid with the exception of two samples, which failed confirmation upon further analysis. All other microbial nucleic acids identified did not meet our relatively rigorous criteria; these were deemed to be environmental contaminants, likely introduced during library preparation or sequencing. Our results therefore support the sterile womb hypothesis at mid-gestation, to the level of detection of this method. This finding is consistent with the notion that amniotic fluid immune responses must not be triggered to avoid early birth, thereby mandating sterility.

To increase confidence in the results generated by our methodology, we developed a set of stringent criteria for pathogen calling: First, we defined a diagnostic read as one in which both paired end reads identify the same species and also required at least three discrete paired end reads to match the same species. Second, we calculate a match score to facilitate removal of human reads that partially match pathogens. This score also allows us to capture pathogen sequences with partial human homology, thus increasing sensitivity. Third, we calculate a Water Normalization Score (WNS) and use a conservative WNS cut-off of 1.0 so that only pathogens found more commonly in samples than in water controls are reported. This is a conservative cut-off because the competing DNA and cDNA from a sample would be expected, probabilistically, to reduce the likelihood of incorporating small amounts of contaminant nucleic acid into the NGS library by one to two orders of magnitude. Fourth, we attempted to confirm all identified pathogens by organism-specific PCR sequencing. We present data that suggests our method is able to detect *E.coli* at < 100 genome copies (gc) per mL amniotic fluid (Suppl Fig. 2C), five different viruses at a level of 1000 gc per mL or lower, and parvovirus B19 at a level of 5000 gc per mL amniotic fluid (Fig. 3A). The method is robust enough to detect the smallest known human pathogen, HDV, which has a 1.68 kb, single stranded RNA, 70% AT rich, genome.

Our findings in mid-gestation concur with those of Lim et al^25^ who studied 24 healthy pregnancies with intact membranes at term. They performed deep sequencing of 16S variable region 4 to look for bacterial nucleic acid, used sequence independent amplification to look for RNA viruses and performed multiple displacement amplification to assess for DNA viruses. They found evidence of one common RNA virus (found in 2–4% of blood donors) in one sample and a bacteriophage in another sample. The total number of 16S reads and the OTU complexity did not differ from buffer controls. Thus, their assessment of the virome and microbiome found amniotic fluid at term to be essentially sterile. Similar results were found using 16S sequencing in other cohorts of 381 pregnancies in mid-gestation^23,26^ and 10 healthy pregnancies at term^24^.

Collectively, our data suggest that positive metagenomic findings in human mid-gestational amniotic fluid are more likely to be due to either noise effects or read mis-assignment. There are several additional possible reasons for the discrepancy between our findings and previous studies that have apparently refuted the sterile womb hypothesis^24,25,57–59^. First and foremost are some of the methodologic concerns we highlight throughout this manuscript. Another possibility is that contamination arose from a number of known sources—environmental, nucleic acid isolation methods, reagents and sequencing equipment—which requires extensive effort to uncover^22,31^. Mishra et al. documented bacteria by culture and scanning electron microscopy in multiple fetal organs from mid-gestational abortuses but did not assess or control for the vaginal microbiome, which was the route of delivery^59^. If in fact fetuses have a way to traffic small numbers of microorganisms to specific organs to enable fetal immune-priming, while simultaneously preventing establishment of a productive infection, the results of Mishra et al. and our studies are not in conflict, which is an intriguing possibility. The only two studies that found significant numbers of mostly skin or vaginal commensal organisms in amniotic fluid used the same SMRT cell technology, so method of library preparation and sequencing platform used may also influence results^57,58^. Alternately, if the time between amniotic fluid collection and freezing is delayed then very small numbers of environmentally introduced commensals may have time to proliferate ex vivo, which is the reason we limited this delay to less than 1 min. Finally, false positive microbial identification can result because pathogen datasets contain human contamination and because commonly used software for taxonomic assignment incorrectly identifies a minority of the human reads as microbial^60^, a problem that led us to develop a taxonomic assignment algorithm as well as cleaned, curated pathogen databases.

Our study has several limitations. First, many unsuccessful attempts were made to incorporate the Avocado Sunblotch Viroid, a 246-nucleotide single stranded circular RNA non-enveloped plant viroid, into NGS libraries, suggesting this class of plant pathogens will not be detected. Second, only six different agents, five viruses and one bacterial species, were tested for detectability out of the several hundred pathogens known to infect humans; however, the effort and cost to determine sensitivity and specificity for all known pathogens would be prohibitive. Third, this method appears suitable for detecting pathogens at low levels but is not useful for generating full coverage genome sequence. Fourth, in amniotic fluid samples with significant white blood cell contamination, the introduction of competitor DNA would be expected to reduce the sensitivity of this method and further studies are needed to establish the magnitude of this effect^27,56^. Finally, our results suggest that amniotic fluid is sterile in mid-gestation, to the level of detection of our assay, but this does not preclude the entry of microbiologic agents later in gestation.

There are many disorders of pregnancy in which an occult viral, protozoan or bacterial infection could be hypothesized to be responsible for a subset of cases, or could serve as an exacerbating factor. As the sensitivity and specificity of metagenomics methods improves, it will become possible to test all of these hypotheses in a definitive manner.

## Supplementary Information

Supplementary Information.

Supplementary Table S3.

Supplementary Table S4.

Supplementary Table S5.

Supplementary Table S6.

Supplementary Table S7.

Supplementary Table S8.

Supplementary Table S9.
