# Supplementary material for: Comprehensive human amniotic fluid metagenomics supports the sterile womb hypothesis
Source: Sci Rep. 2022 Apr 27;12:6875. doi: 10.1038/s41598-022-10869-7 (PMC9046152; doi:10.1038/s41598-022-10869-7)
Supplement: Supplementary file 1 — Supplementary Information. [file 41598_2022_10869_MOESM1_ESM.pdf]

SUPPLEMENTAL MATERIALS

Comprehensive human amniotic fluid metagenomics supports the sterile womb hypothesis

HanChen WANG, MSc[1,2]; Gui Xiang YANG, DVM[1]; Yuxiang HU, MD[3,4]; Patricia LAM, PhD[1,5]; Karan SANGHA, MSc[1]; Dawn SICILIANO, MSc[1]; Anne SWENERTON, MSc[1]; Ruth MILLER, PhD[6,7,8]; Peter TILLEY, MD[6,7]; Peter VON DADELSZEN, MBChB DPhil[3,9]; Shirin KALYAN, PhD[10]; Patrick TANG, MD PhD[6,7,11]; Millan S. PATEL*, MD MSc[1,12]

[1] Department of Medical Genetics, University of British Columbia, Vancouver, BC, Canada
[2] Department of Physiology, McGill University, Montreal, QC, Canada
[3] Department of Obstetrics & Gynaecology, University of British Columbia, Vancouver, BC, Canada
[4] CureImmune Therapeutics Inc., Vancouver, BC, Canada
[5] Center for Gene Therapy, Abigail Wexner Research Institute, Nationwide Children's Hospital, Columbus, OH, USA
[6] Department of Pathology and Laboratory Medicine, University of British Columbia, Vancouver, BC, Canada
[7] British Columbia Centre for Disease Control, Vancouver, BC, Canada
[8] Contextual Genomics Inc., Vancouver, BC, Canada
[9] Department of Women and Children's Health, School of Life Course Sciences, King's College London, London, United Kingdom
[10] Department of Medicine, Division of Endocrinology & Metabolism, University of British Columbia, Vancouver, BC, Canada
[11] Department of Pathology, Sidra Medical and Research Center, Doha, Qatar
[12]BC Children's Hospital Research Institute, University of British Columbia, Vancouver, BC, Canada
The authors report no conflict of interest.

Content **Pages**

Supplemental Figures and Figure Legends S1-S3 2-7
Supplemental Table Legend 8-9

SUPPLEMENTAL FIGURES

Suppl Figure 1. Optimizing thread number for MEGABLAST detection. The elapsed time of MEGABLAST detection to the human genomic sequences found in NCBI nt database (one of the host genomic sequence removal steps in our pipeline) were calculated for fifteen groups of 10,000 sequences randomly selected from the amniotic fluid samples. The detection was performed on CentOS6 with Linux kernel version 2.6.32 using NCBI blast 2.2.30+ version with up to twenty-four Intel(R) Xeon(R) CPU X5650 at 2.67 GHz processors.

Suppl Figure 1

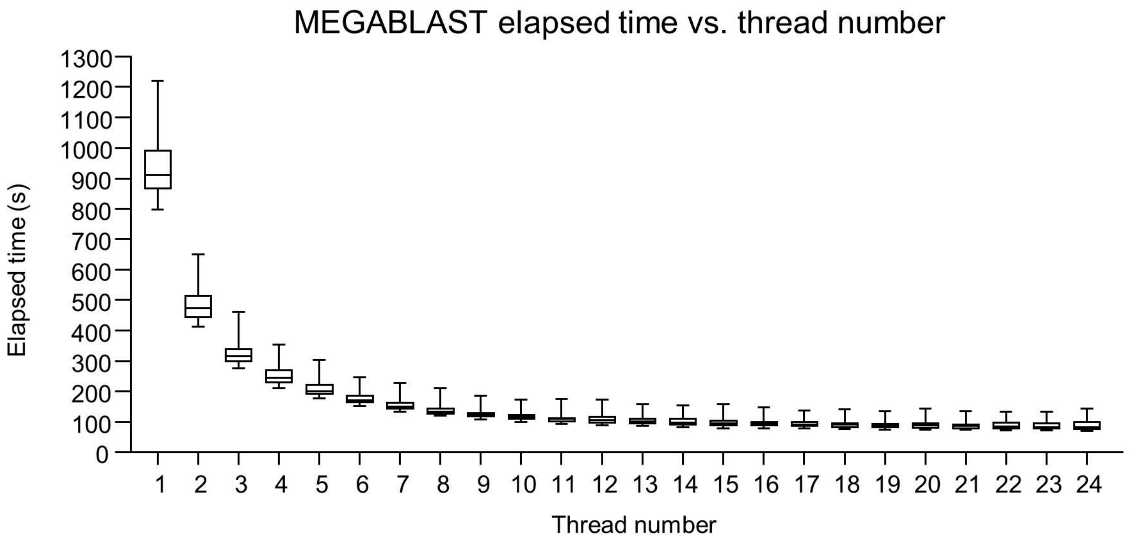

Suppl Figure 2. Comparison of yield of the smallest human pathogen, HDV (**A**), and one of the largest, CMV (**B**), viruses by 4 extraction methods: 1) Ambion kit, 2) Phenol/Trizol, 3) Qiagen kit, and 4) Zymo kit. White bars indicate a 10,000 genome copy (gc) spike, grey bars a 1,000 gc spike and black bars a 100 gc spike. Ordinates are log scale. (**C**) Number of correct sequence reads obtained after spiking 228 gc of Enterovirus B, 385 gc of HDV and 22 *E. coli* into control amniotic fluid. Library total: 141 million reads. (**D**) Dot plot showing the degree of homology, as assessed by BLASTn, between 16S rDNA sequences from cloning and sequencing and the cognate regions from IDSeq results with at least 100 hits for a species. Source data for this panel is in Suppl Table 8.

Suppl Figure 2

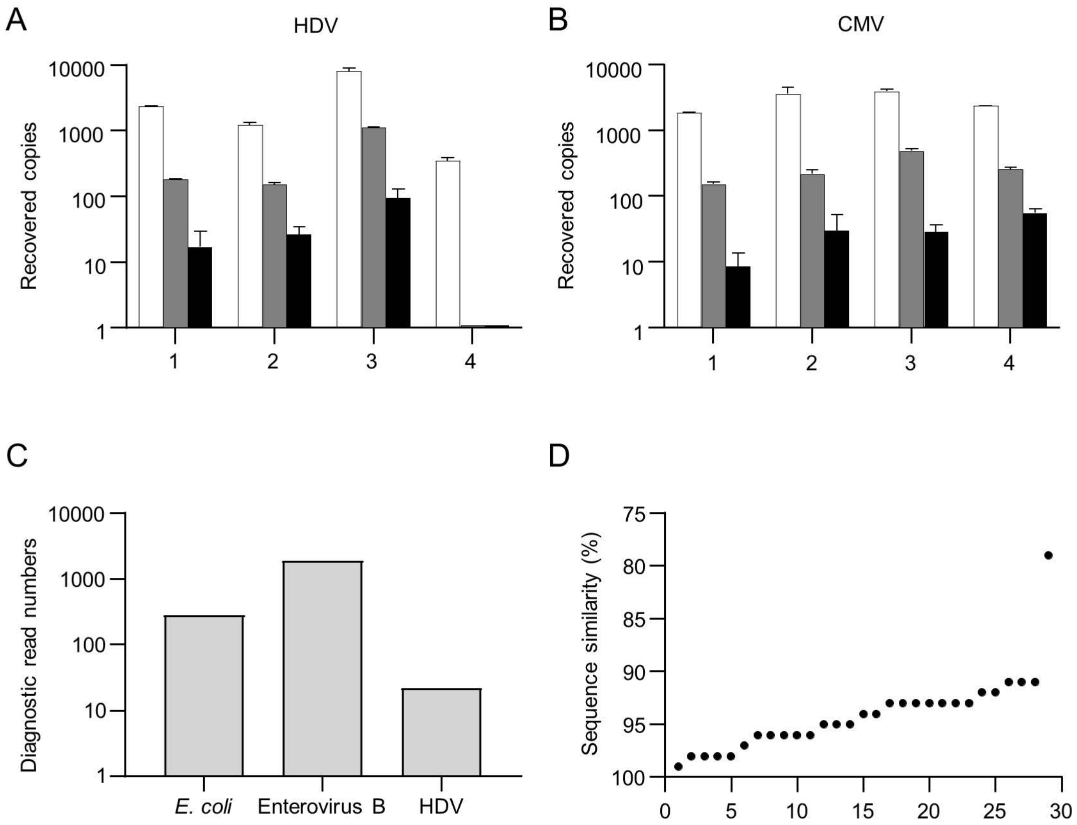

Suppl Figure 3. Minimal run to run variation in spectrum of species detected. The fraction of the 10 most common organisms found in two water samples (left panel) or two amniotic fluid samples (right panel) from two different runs and up to 6 different lanes of a flow cell is shown.

Suppl Figure 3

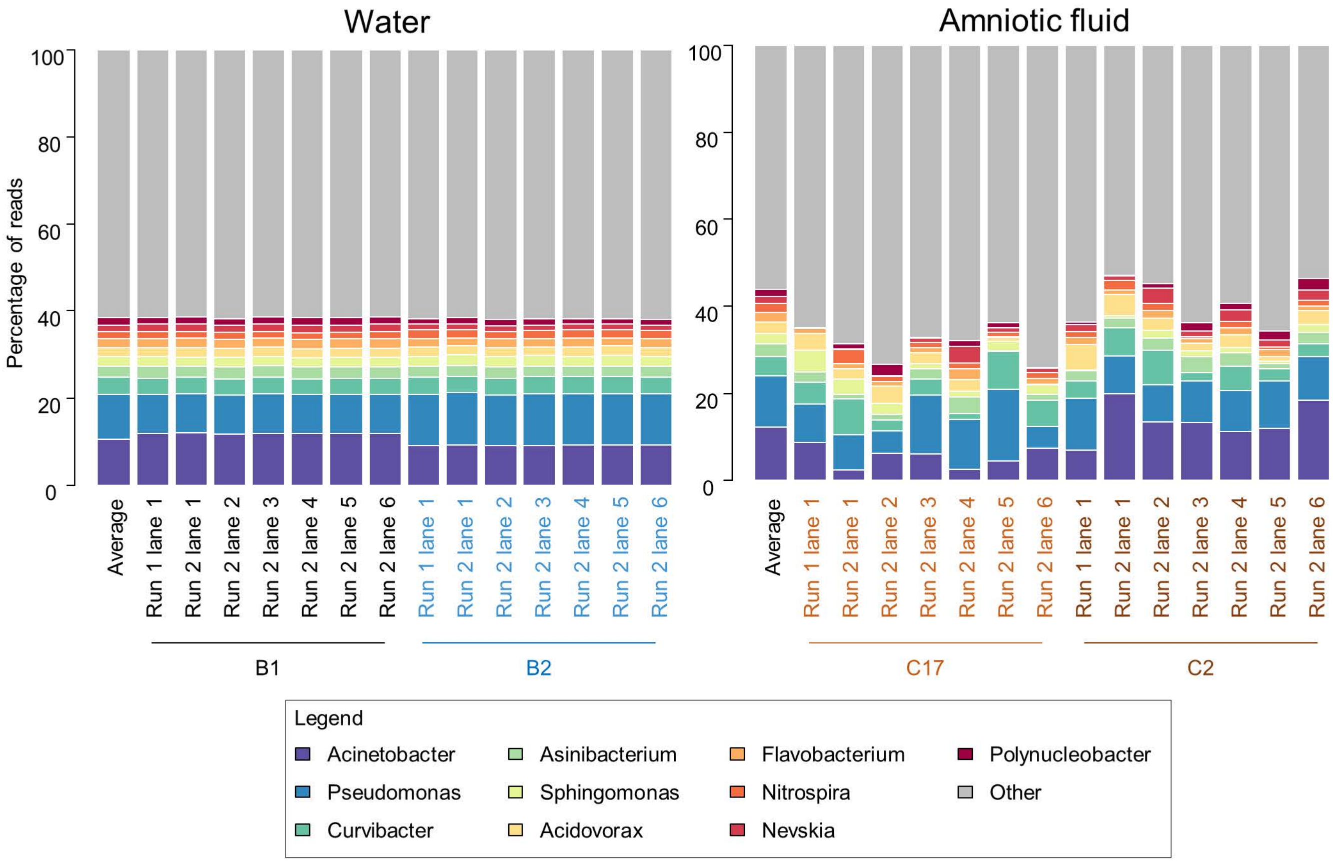

SUPPLEMENTAL TABLE LEGEND

Suppl Table 1 – Method optimization: Fraction of genome copies recovered after second strand synthesis

Species	After cDNA synthesis	After second strand synthesis
CMV<br>Taxon ID: 10359	0.57	0.33
Enterovirus B<br>Taxon ID: 138949	2.81	0.74
HDV<br>Taxon ID: 12475	0.25	0.20
PhiX174<br>Taxon ID: 2607483	0.21	0.17

Suppl Table 2 – Method optimization: Fold recovery of genome copies recovered at each step of library synthesis

Species	After Qiagen column	After cDNA synthesis	After Zymo column	After library amplification	After library purification
CMV<br>Taxon ID: 10359	0.78	0.057	0.078	54.8	41.7
Enterovirus B<br>Taxon ID: 138949	-	0.606	0.583	164.3	131.0
HDV<br>Taxon ID: 12475	-	0.027	0.106	24.9	14.4
PhiX174<br>Taxon ID: 2607483	0.58	0.054	0.006	2.5	1.0

Suppl Table 3 – Counts of organisms with removed sequences
See appended spreadsheet.

Suppl Table 4 – Coordinates of sequences removed from viral reference sequences due to human homology
See appended spreadsheet.

Suppl Table 5 – Coordinates of sequences removed from bacterial reference sequences due to human homology

See appended spreadsheet.

Suppl Table 6 – Coordinates of sequences removed from protozoan reference sequences due to human homology

See appended spreadsheet.

Suppl Table 7 – List of primers used

See appended spreadsheet.

Suppl Table 8 – Detection by IDSeq vs 16S rDNA sequencing

See appended spreadsheet. Each 16S rDNA clone sequenced was matched to the closest species identified by IDSeq with at least 100 hits. The percent sequence similarity of the 16S sequence to the IDSeq species 16S was calculated by subtracting all mismatches and gaps from the number of identical nucleotides and dividing that by the total alignment length.

Suppl Table 9 – Read numbers at different filtering stages

See appended spreadsheet.
